# MMV in partnership: the Eurartesim^®^ experience

**DOI:** 10.1186/1475-2875-12-211

**Published:** 2013-06-19

**Authors:** David Ubben, Elizabeth M Poll

**Affiliations:** 1 Medicines for Malaria Venture, 20, rte de Pré-Bois, PO Box 1826, Geneva 1215, Switzerland

## Abstract

**Background:**

This case study describes how a public-private partnership between Medicines for Malaria Venture (MMV) and Sigma-Tau Industrie Farmaceutiche Riunite SpA achieved international regulatory approval for use of the fixed-dose artemisinin-based combination therapy dihydroartemisinin-piperaquine (Eurartesim^®^) for the treatment of malaria, enabling more widespread access to the medicine in malaria-endemic countries.

**Case description:**

The combination of dihydroartemisinin and piperaquine demonstrated success in clinical trials for the treatment of malaria in Asia and Africa in the 2000s. However, as it had not been developed to international regulatory standards it was out of the reach of the majority of patients in disease-endemic countries, particularly those reliant on public healthcare systems supported by international donor funding. To overcome this, as of 2004 MMV worked in partnership with Sigma-Tau, Holleykin, Oxford University, the Institute of Tropical Medicine Antwerp, and the National Institute of Malaria Research India to develop the dihydroartemisinin-piperaquine combination to international standards. In 2011, the European Commission granted full marketing authorization to Sigma-Tau for Eurartesim*.*

**Discussion and evaluation:**

The partnership between MMV, Sigma-Tau, and numerous other academic and industrial partners across the world, led to the successful development to EMA regulatory standards of a high-quality and highly efficacious anti-malarial treatment that otherwise would not have been possible. The dossier has also been submitted to the WHO for prequalification, and a safety statement to guide correct use of Eurartesim has been produced. In July 2012, the first delivery to a disease-endemic country was made to Cambodia, where the medicine is being used to treat patients and help counter the emergence of artemisinin resistance in the area. A paediatric dispersible formulation of Eurartesim is being developed, with the objective to submit the dossier to the EMA by the end of 2014.

**Conclusions:**

The development of Eurartesim to international regulatory standards exemplifies the strengths of the product development partnership model in utilising the individual skills and expertise of partners with differing objectives to achieve a common goal. Successful uptake of Eurartesim by public health systems in malaria-endemic countries poses new challenges, which may require additional partnerships as we move forward.

## Background

Eurartesim^®^ (dihydroartemisinin-piperaquine phosphate; DHA-PQP) is an artemisinin-based combination therapy (ACT) that received regulatory approval from the European Medicines Agency (EMA) on 27 October 2011. With this high-quality stamp of approval in hand, the medicine is now being used safely and effectively and can be brought to many more malaria patients than earlier versions of the same combination. In July 2012, the first shipment of Eurartesim was despatched in Cambodia, where its use will help to stem the development of artemisinin drug resistance emerging in the region. This case study describes the partnership between Medicines for Malaria Venture (MMV) and Sigma-Tau Industrie Farmaceutiche Riunite SpA, and ultimately many other partners, that enabled this objective to become a reality.

Malaria places a huge burden on disease-endemic countries of the developing world, both economically and in terms of human suffering. High-quality affordable anti-malarial drugs must continue to be developed to ensure that patients receive adequate treatment and to slow the development of drug resistance. The anti-malarials market in disease-endemic countries is flooded with poor quality or counterfeit medicines, often with fatal repercussions for patients, and so quality is key [1]. In recent decades, however, the increased costs of developing and registering pharmaceutical products, coupled with the prospect of inadequate commercial returns, resulted in the withdrawal of the majority of research-based pharmaceutical companies from research and development (R&D) investment in malaria. Moreover, public sector government agencies and the not-for-profit private sector are not individually equipped with all the skills and resources necessary to discover, develop, and commercialize new anti-malarial drugs.

International realization of the deteriorating situation with regard to the discovery and development of new high-quality treatments for malaria and other neglected diseases in developing countries led to the emergence of the public-private product development partnership (PDP) model. In 1999, specifically for malaria, the model was brought to life in the form of a not-for-profit partnership, MMV that receives funding from governments and philanthropic organizations. The PDP model, as applied by MMV, offers an alternative approach to conventional drug discovery and development that brings together partners from the public sector (e.g., government agencies and international institutions such as the World Health Organization [WHO]), the for-profit private sector (e.g., pharmaceutical and biotechnology companies), and civil society (e.g., academia, the not-for-profit private sector, and philanthropic institutions). MMV’s strength lies in the complementarity of skills and resources provided by its individual partners, its extensive communication networks, and in-house malaria R&D expertise, which accelerate the discovery, development, and delivery of new medicines.

PDPs such as MMV frequently do not have the infrastructure themselves to undertake research and development projects in-house. Instead, they work in partnership with institutions or companies with existing facilities and expertise to conduct research, such as laboratories or clinical sites. The partners provide in-kind contributions and/or financing to their specific projects, while PDPs manage the complete project portfolio, focusing on allocating resources, inputting scientific expertise and coordinating partner activities throughout the complete R&D process. MMV manages a complete project portfolio, bringing dedicated sources of funding and expertise to committed researchers to enable the discovery, development and delivery of new medicines for malaria.

MMV has forged over 287 successful partnerships across almost 50 countries since its inception. Today, the project portfolio includes approximately 65 anti-malarial projects. The WHO-recommended treatment for uncomplicated *Plasmodium falciparum* malaria is fixed-dose ACT [2]. In line with this recommendation in 2004 a partnership was formed between MMV, Holleykin (comprising the Chinese Holley group and Guangshou University of Traditional Chinese Medicine), the Italian pharmaceutical company Sigma-Tau Industrie Farmaceutiche Riunite SpA and Oxford University. The objective was to develop and gain international regulatory approval for use of the fixed-dose ACT DHA-PQP (brand name Eurartesim^®^). The combination of DHA-PQP (under other brand names from other manufacturers) had been used widely and demonstrated usefulness as a key treatment against malaria in the private sector in certain countries in Asia and Africa [3-8]. With the emergence of resistance to artemisinin and its derivatives in south-east Asia, WHO’s Global plan for artemisinin resistance containment (GPARC) calls for improved access to quality-assured diagnostics and treatment with ACT [9]. Since 2008, DHA-PQP has been the first-line drug of choice in Cambodia, one of the key countries leading the fight to contain artemisinin-resistant strains of malaria [10]. However, the EMA approval of Eurartesim in 2011, the public sector had no access to it as it had not been approved by a stringent regulatory authority and thus could not be procured using international donor funds. MMV and Sigma-Tau worked together to develop the combination to high regulatory standards in order to change that and help bring the medicine to more vulnerable patients.

## Case description

### Early development of dihydroartemisinin-piperaquine

**Figure 1:**
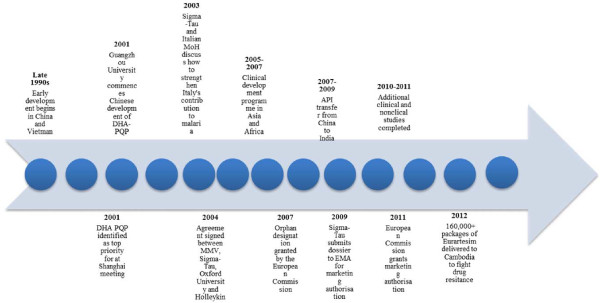
Timeline of key events in the development of Eurartesim^®^ to international regulatory standards.

Initially, PQP and DHA were developed and tested separately as monotherapies. PQP, a bisquinoline anti-malarial, was first synthesized in the 1960s at Rhone-Poulenc in France and, independently, developed and tested at the Shanghai Research Institute of Pharmaceutical Industry in China. Due to the increasing prevalence of chloroquine (CQ)-resistant parasites in China, PQP was adopted as the first-line treatment between 1978 and 1994 [11]. PQP monotherapy was used broadly for both malaria prophylaxis and treatment, with the equivalent of 140 million adult doses manufactured and distributed [12]. However, resistance to PQP began to emerge in the late 1980s onward [13]. At the same time, in Thailand, it was discovered that by combining mefloquine, which had largely failed as monotherapy due to resistance, with artesunate, it was possible to restore effective anti-malarial activity. Such a combination also required less artesunate than monotherapy with artesunate – an expensive and rare drug at the time. These factors, together with the desire to have a presence in the international pharmaceutical industry, sparked the interest of the Chinese scientific community in developing ACT, and they put a number of combinations to the test (Figure 1).

Professor Li Guoqiao of Guangzhou University of Traditional Chinese Medicine initially developed the combination of DHA-PQP in the late 1990s. At that time, his goal was to develop a highly affordable medicine, and so he opted for the less expensive DHA rather than artesunate; its production involves one synthetic step less than artesunate, and it is, therefore, more cost-effective yet still very efficacious against malaria. In 2001, clinical development of the drug combination commenced through a collaboration with Professor Tran Tinh Hien of the Hospital for Tropical Diseases (HTD) and the Wellcome Trust Major Overseas Programme in Vietnam. An important randomized controlled study of a fixed-dose combination of DHA-PQP was undertaken at the HTD, and the drug combination was found to be safe and highly efficacious [6].

Following Professor Hein’s trial, other clinical investigations of DHA-PQP were also carried out in Thailand and Cambodia, all confirming the excellent safety and efficacy of the combination [3-5,7].

In 2002, the Holley group, a Chinese company involved in the production of artemisinin-based anti-malarial drugs, formed a joint venture with Guangzhou University of Traditional Chinese Medicine named Holleykin. This brought the DHA-PQP combination product into the Holley group portfolio. Clinical development work continued on DHA-PQP, and in 2003, the product achieved registration in China under the name of Artekin^®^. The name, however, was later abandoned in favour of Eurartesim, owing to its similarity to the name of another anti-malarial combination treatment, Artequin^®^, which contained artesunate and mefloquine.

### The first contacts – East meets West

The combination of DHA-PQP caught the attention of the WHO in early 2001 [2]. Later that year, MMV was invited by the WHO Western Pacific Regional Office to participate in a meeting convened in Shanghai on how best to facilitate uptake of DHA-PQP and other forms of ACT by public health systems in malaria-endemic countries. The United States Pharmacopeia (USP) was also invited, and other meeting participants included members of the scientific community and Chinese government, as well as representatives of Chinese commercial interests. Through the highly successful use of dihydroartemisinin, piperaquine, primaquine, and trimethoprim combined into one tablet in Vietnam in the 1990s (under the names China-Vietnam drug number 4 and 8; CV4/CV8 or Artecom^®^), the DHA-PQP combination had already demonstrated significant potential as an anti-malarial treatment capable of positively impacting public health and reducing the malaria burden [6,14,15]. MMV recognized this potential. Participants of the 2001 Shanghai meeting concluded that although DHA-PQP demonstrated significant efficacy against malaria, its production using Chinese Good Manufacturing Practice (GMP) laws at that time did not match the regulatory standards of Europe and the USA. This meant that further development of the product would be needed in order to meet international regulatory standards to facilitate its uptake by public health systems in malaria-endemic countries. International regulatory approval would allow the product to be purchased using international donor funds. At the time, Chinese GMP bore a number of differences to EU and US GMP in terms of the level of detail of its quality control requirements, meaning that specific implementation of the requirements could significantly vary between production plants. (Chinese GMP has since evolved, and the new Chinese GMP 2010 (effective since March 1, 2011) contains many more detailed requirements on key aspects of the drug manufacturing process, bringing the key provisions of Chinese GMP into alignment with EU and US GMP [16].)

Drawing on its strong regulatory expertise, MMV proceeded to hold discussions with Holleykin about how best to develop DHA-PQP to international standards. In 2003, a non-disclosure agreement was signed between MMV and Holleykin to work towards this goal. MMV commenced an analysis of the elements that would be necessary for a potential dossier on DHA-PQP suitable for submission to an international regulatory authority.

The USP Drug Quality and Information Program was also engaged at this time to examine the GMP capabilities of the Guangdong Medi-World Pharmaceutical Company, responsible for manufacturing Artekin within the Holley group. They considered that it should be possible for the pharmaceutical company to establish sufficient controls and capabilities to consistently manufacture the product according to international GMP standards, without any major upgrade of the existing facilities.

### The partnership comes together

In 2003, Dr Claudio Cavazza (Founder and then President of Sigma-Tau) and Dr Girolamo Sirchia (then Italian Minister of Health) held discussions about the importance of continuing Italy’s scientific contribution in the field of malaria and possible ways this could be achieved. Dr Sirchia suggested the idea of a collaboration between Sigma-Tau, MMV, Holleykin, and the University of Oxford (UK). The University of Oxford (through its unit in Bangkok, the Mahidol Oxford Tropical Medicine Research Unit [MORU]) had played a leading role in researching ACT and had done some pioneering work on the combination of DHA/PQP from China [17]. Contact was made between Sigma-Tau and the Holley group, facilitated by representatives of the WHO Tropical Disease Research programme and MMV. In 2004, a four-way agreement was signed between Sigma-Tau, the Holley group, Oxford University and MMV to develop a DHA-PQP combination product to international quality standards.

The partnership also drew on MMV’s extensive network of world-class partners, including the Institute of Tropical Medicine Antwerp (Belgium) and the National Institute of Malaria Research (NIMR) in India. All partners worked towards the same goal of developing DHA-PQP to stringent standards in order to gain international regulatory approval for the product. The Oxford scientists were able to provide a wealth of clinical background and know-how on conducting clinical trial in malaria endemic countries and of DHA-PQP, as they had worked with the combination for a number of years through their south-east Asian collaborations. They were also able, in addition to the scientists from Antwerp and NIMR, to offer access to their dedicated clinical trial sites in Asia and Africa to advance the development process, which were considered centres of excellence. This included sites in Thailand, Laos, and Kenya.

Both MMV and Sigma-Tau possessed different skill sets and expertise, which enabled them to maximize their individual strengths in a complementary manner. MMV was able to provide strong regulatory and clinical expertise and guidance, as well as in-house experience in malaria R&D [18], while Sigma-Tau possessed high-level fully compliant European GMP pharmaceutical laboratories and production facilities, as well as expertise in drug development.

In 2006, recognising the threat of drug resistance to artemisinin, the gold standard anti-malarial treatment, the WHO urged governments to ban the use of artemisinin monotherapy [19]. At that time, the Holley group’s sole source of pharmaceutical revenue came from an artemisinin monotherapy product named Cotecxin^®^. When faced with the WHO demand, the Holley group agreed to progressively stop producing its DHA monotherapy. However, the group had to move quickly to replace this product and subsequently pulled out of the agreement to develop Eurartesim and commercialized their existing version of DHA-PQP, Duo-Cotecxin^®^.

MMV and Sigma-Tau remained in partnership and continued their effort by first examining the existing quality, clinical, and non-clinical data on DHA-PQP held by the Holley group. It became clear that much of the existing product data would be insufficient for the requirements of a stringent regulatory authority; data had been collected from diverse drug production batches of differing quality and from diverse research settings, and the active compounds originated from a variety of sources and had been manufactured using different methods. Moreover, little of the available documentation could have been considered to have been produced under the internationally recognized conditions of GMP, Good Laboratory Practice, and Good Clinical Practice.

### The development plan

MMV and Sigma-Tau worked together to create a clinical development plan able to deliver all the necessary data for submission of a dossier on Eurartesim to a stringent regulatory authority. They drew on the malaria expertise of Oxford and the Institute for Tropical Medicine in Antwerp, while using the tools of modern drug development to maintain the highest regulatory standards. The partnership jointly determined the timeframes for each section of the development plan, while working together with scientists who had previously tested the combination to discover the optimal dosing schedule. They drafted a clinical development plan, which included three pharmacokinetics studies and two pivotal Phase III randomized trials (Table 1).

**Table 1:** Studies of Eurartesim in patients

**Study**	**Dose**	**Test regimen**	**Comparator**	**Total no. of subjects**	**Subject type**	**Main efficacy endpoint**	**Countries**
ST3073+ST3074 DM09006 Phase I	Dose range: 1.6-2.5 mg/kg/day DHA and 12.4-20.3 mg/kg/day PQP	40 mg DHA/320 mg PQP tablet. 3 oral doses in 48 hrs.	Placebo, Riamet,(AL), moxifloxacin	268	Adult healthy volunteers	To evaluate the effect of multiple oral doses of DHA and PQP on the QT/QTc interval compared to AL, placebo and moxifloxacin in healthy male and female volunteers	France
ST3073+ST3074 DM09007 Phase I	Dose range: 1.6-2.7 mg/kg/day DHA and 12.4-21.8 mg/kg/day PQP	40 mg DHA/320 mg PQP tablet. 3 oral doses in 48 hrs.	Not Applicable	72	Adult healthy volunteers	DHA and PQP PK in healthy male and female Asian and Caucasian volunteers after single and repeated dose of Eurartesim	Australia
ST3073+ST3074 DM09008 Phase I	Dose range: 1.7-2.1 mg/kg/day DHA and 13.9-17 mg/kg/day PQP	40 mg DHA/320 mg PQP tablet. One single oral dose.	Not Applicable	37	Adult healthy volunteers	To assess the effect of food on the PK of DHA and PQP after single oral administration of Eurartesim in healthy male adult volunteers	Australia
ST3073+ST3074 DM 04008 (Africa) Phase I/II	Dose range: 1.67-3.08 mg/kg/day DHA and 13.3-24.64 mg/kg/day PQP	20 mg DHA/160 mg PQP or 40 mg DHA/320 mg PQP tablet. 3 oral doses in 48 hrs.	Not Applicable	32	Paediatric *P. falciparum* malaria patients	To assess the PK of DHA and PQP by analysing serial blood samples during and after a therapeutic course in children with malaria	Burkina Faso
ST3073+ST3074 DM04009 (Asia) Phase I/II	Dose range: 1.6-3.64 mg/kg/day DHA and 12.8-29.12 mg/kg/day PQP	40 mg DHA/320 mg PQP tablet. 3 oral doses in 48 hrs.	Not Applicable	25	Adult *P. falciparum* malaria patients	To assess the pharmacokinetics of DHA and PQP by analysing serial blood samples during and after a therapeutic course in adults with malaria.	Thailand
ST3073+ST3074 DM040010 (Asia) Phase III	Dose range:1.67-3.33 mg/kg/day DHA and 13.3-26.7 mg/kg/day PQP	20 mg DHA/160 mg PQP or 40 mg DHA/320 mg PQP tablet. 3 oral doses in 48hrs.	50 mg AS + 250 mg MQ AS 4 mg/kg/day for 3 days + MQ 25 mg/kg divided into two doses	1150	Adult and paediatric *P. falciparum* malaria patients (even in combination with other Plasmodia)	To demonstrate that the PCR-corrected cure rate of DHA-PQP at Day 63 is non-inferior to that of AS+MQ (non-inferiority margin=5%)	Thailand, India, Laos
ST3073+ST3074 DM040011 (Africa) Phase III	Dose range: 1.6-3.64 mg/kg/day DHA and 12.8-29.12 mg/kg/day PQP	20mg DHA/160mg PQP or 40mg DHA/320 mg PQP tablet. 3 oral doses in 48 hrs	20 mg A/120 mg L 2-6 tablets per day over 3 days dependant on body weight	1553	Paediatric *P. falciparum* malaria patients	To demonstrate that the PCR-corrected cure rate of DHA-PQP at Day 28 is non-inferior to that of AL (non-inferiority margin=5%)	Burkina faso, Kenya, Uganda, Mozambique, Zambia

Clinical trial supplies were produced by Sigma-Tau at their European GMP-compliant plant in Pomezia, Italy, initially using active pharmaceutical ingredients (APIs) from the Holley group in China. A plan was then executed to qualify the API and develop acceptable specifications, as well as to minimize impurities, and, where needed, to perform regulatory non-clinical qualification of them. Sigma-Tau and MMV eventually took the decision to move production of the APIs from China to the Indian pharmaceutical company Arch Pharmalabs. Arch Pharmalabs possessed current GMP-compliant manufacturing facilities that had been inspected and approved by global regulatory agencies.

The pharmacokinetics of piperaquine were largely unknown in 2001. As the drug was known to have an extremely long half-life, it was decided that it would be important to carry out stringent analysis of the pharmacokinetic profile of DHA-PQP in different populations. This served the purpose of defining the possibility of accumulation of piperaquine, which might be considered a safety and regulatory risk for the combination. As it turned out, the information that was gathered proved vital in achieving the registration later on. The clinical pharmacokinetics studies conducted were as follows: (i) a Phase I single-dose study carried out in 2005 in Switzerland in healthy volunteers; (ii) a Phase I/II study carried out during 2005 and 2006 in adult patients in Asia; and (iii) a Phase I/II study carried out in 2006 in paediatric patients in Africa.

### Eurartesim clinical trial programme

Two pivotal Phase III trials were carried out in Asia and Africa, respectively, to account for the differences in baseline immunity and transmission risks for malaria in differing endemic regions. Both were multicentre, randomized, open label, two-arm, parallel group studies. The Asian study was conducted between June 2005 and April 2007 at 10 study sites across India, Laos, and Thailand, with the objective of determining whether DHA-PQP was non-inferior to the loose combination of artesunate + mefloquine as well as assessing the efficacy and safety of DHA-PQP in Asian patients with acute uncomplicated *P. falciparum* malaria [20-22]. The main efficacy measure was the proportion of patients who were cured 63 days after treatment (determined using polymerase chain reaction (PCR) genotype-correction). The African study was conducted between August 2005 and July 2006 at five study sites in Burkina Faso, Kenya, Mozambique, Uganda, and Zambia. The objective of the study was to determine whether DHA-PQP was non-inferior to artemether-lumefantrine and to assess its safety and tolerability in African children with acute uncomplicated *P. falciparum* malaria [23,24]. The main efficacy measure was the proportion of patients cured 28 days after treatment, (determined using PCR genotype-correction). Patients were, however, followed up to day 42 as a secondary endpoint. In total, 2,703 patients were included in the Phase III studies.

Both Phase III studies were guided by a clinical development committee that primarily addressed the scientific conduct, ethical integrity, and regulatory acceptability of the trial programme [25]. The clinical development committees each comprised one representative from MMV, two representatives from Sigma-Tau, and the study’s Coordinating Investigator. In addition, both studies included a data monitoring committee responsible for performing an interim analysis for sample size reassessment, monitoring safety data during the study, and revising the primary efficacy data [25]. The data monitoring committees each included one statistician and four clinicians with malaria expertise who were independent of Sigma-Tau and MMV.

The results of the Eurartesim clinical trial programme confirmed the product’s safety and high level of efficacy. In the Asian Phase III trial, 97% of patients were cured 63 days after treatment compared with 95% of patients who received the comparator artesunate + mefloquine. Additional, country-specific analysis of the Phase III data from India led the study authors to suggest DHA-PQP could be considered for the first-line treatment in country [26]. In the African Phase III trial, 93% of patients were cured 28 days after treatment compared with 95% of patients treated with artemether-lumefantrine (differences between treatment groups were non-significant and percentages are based on modified intention to treat populations) (Table 2). Both studies also found that the occurrence of new infections during the follow-up period after treatment was significantly lower with DHA-PQP than the comparator treatments, indicating that DHA-PQP has a comparatively longer prophylactic effect after treatment [20,23].

**Table 2:** Results of the two Phase III open-label clinical trials

**Study**	**PCR-corrected cure rate (mITT)**		
	**Eurartesim**	**AS+MQ**	**A+L**
DM040010 (n=1087)	97.0%	95.3%	-
DM040011 (n=1524)	92.7%	-	94.8%

A+L: artemether+lumefantrine; AS+MQ: artesunate+mefloquine; mITT: modified intention-to-treat (defined as all randomized patients who received at least one dose of study treatment, with the exclusion of those patients lost to follow-up for unknown reasons); PCR: polymerase chain reaction.

MMV and Sigma-Tau took the decision not to carry out extensive non-clinical studies with Eurartesim other than the aforementioned pharmacokinetic analysis of piperaquine, on the basis that between 1976 and 1994 the equivalent of 140 million adult doses of piperaquine had been used in China [11,12,27]. As such, PQP would have qualified as a ‘well-established substance’, as defined in the EU regulations [28]. Nonetheless, the documentation for this use proved less than convincing for the EMA. This regulation on well-established substances was then revised in 2007 by the EMA [29] and, therefore, additional non-clinical testing was required.

### Regulatory decisions

Initially, MMV and Sigma-Tau had made the decision to seek Italian regulatory approval for Eurartesim. Sigma-Tau already possessed strong Italian regulatory expertise, so an application for regulatory approval through the national route would enable them to use this expertise. In 2005, an Italian Investigational New Drug application was filed with the National Institute of Health (Istituto Superiore di Sanità), which allowed Phase I studies to begin. After further extensive evaluation drawing upon MMV’s own strong regulatory expertise, a decision was made to apply for European centralized registration via an orphan drug application, rather than Italian national registration. As an orphan drug application, the dossier would benefit from an accelerated review process. Moreover, the regulatory seal of approval from the EMA for Eurartesim as an orphan drug would enable the product dossier to be filed and accepted more quickly in disease-endemic countries. It would also allow Sigma-Tau to market the product in Europe, where they already had a commercial presence. On 3 August 2007, orphan designation (EU/3/07/468) was granted by the European Commission to Sigma-Tau Industrie Farmaceutiche Riunite SpA, Italy, for DHA-PQP for the treatment of malaria.

In July 2009, MMV and Sigma-Tau submitted their completed dossier on Eurartesim to the EMA for marketing authorization. In July 2010, a list of questions was received from the Committee for Medicinal Products for Human Use (CHMP) in response to the submission. Among the questions, the CHMP noted a potential risk for QTc interval prolongation with Eurartesim. Over the next six months, MMV worked closely with Sigma-Tau to produce the response to the CHMP questions, which necessitated three further clinical trials and 25 new non-clinical studies. In collaboration with MMV, Sigma-Tau pulled together all of its resources to work intensively to produce the necessary data and responses to the questions, and in June 2011, the CHMP of the EMA adopted a positive opinion, recommending the granting of marketing authorization in Europe for Eurartesim film-coated tablet for the treatment of uncomplicated *P. falciparum* malaria in adults, children, and infants aged 6 months and over and weighing 5 kg or more. On 27 October 2011, the European Commission granted full marketing authorization in 27 member states, making it the first ACT to be approved by the EMA for the treatment of uncomplicated *P. falciparum* malaria using a centralized procedure.

### Eurartesim product and safety

Eurartesim is generally well tolerated and has a simple dosage regimen that involves weight-based administration once daily (up to four tablets per dose) for three days [30]. This makes the drug more patient friendly and could increase compliance over other currently-available forms of ACT requiring twice-daily administration. Owing to the long half-life of piperaquine, Eurartesim also provides better and longer protection from new malaria infections than other forms of ACT, as demonstrated during its clinical development programme [20]. This is an important advantage, particularly for children in high transmission areas who are often affected by a second life-threatening malaria episode after recovering from their original infection [23]. Moreover, the formulation of Eurartesim means that it has a two-year shelf life in disease-endemic countries. A recent study reported Eurartesim to be the most stable form of the six DHA-based formulations investigated [31]. Eurartesim is available in two different packs (one for adults and one for children) with different quantities of the active ingredients in the tablets: each film-coated tablet contains either 160 mg piperaquine tetraphosphate (as the tetrahydrate; PQP) and 20 mg DHA, or 320 mg PQP and 40 mg DHA [30].

Following EMA registration, MMV and Sigma-Tau worked together to produce a safety statement on Eurartesim to guide correct prescription of the product [see Additional file 1 for complete safety statement]. The CHMP considered that the current safety database on Eurartesim was not sufficiently large to determine whether the QTc effect of Eurartesim would translate into arrhythmias, and if so, how frequently these might occur (although they did consider that the non-clinical data suggested the torsadogenic potential of Eurartesim is lower than chloroquine and similar to artemether-lumefantrine) [32]. To enable the optimal deployment and use of Eurartesim, a repeat-dose study is underway in West Africa (The West African Network for Clinical Trials of Antimalarial Drugs: WANECAM) and a safety and effectiveness study is due to commence in Ghana, Burkina Faso, Mozambique and Tanzania in 2013 (INDEPTH Effectiveness and Safety Studies of Antimalarials in Africa: INESS).

## Discussion and evaluation

This case study illustrates how a strong PDP collaboration between partners with diverse skills, expertise, and motivations can lead to success. The development of Eurartesim to international regulatory standards, thereby enabling the medicine to reach many more patients, would simply not have been possible without these partnerships. Although the drug combination DHA-PQP was already in existence and available in the private sector, and had been used extensively in China, Vietnam, and certain other countries in Asia, the formulation had not previously been manufactured according to internationally recognized GMP. Consequently, it could not be purchased with international donor funding and therefore accessed by the public health systems of the majority of malaria-endemic countries in the developing world.

Recognition of the potential of DHA-PQP to positively impact public health in malaria-endemic countries initially arose at the WHO and through visionary national malaria control programmes such as that of the Vietnamese HTD. A strong partnership existed between MMV and health experts at the WHO, which meant that MMV was in a position to collaborate with the WHO at an early stage to explore the potential of DHA-PQP. MMV was also able to draw on the input of other world-class partners during the clinical development programme, including researchers from the University of Oxford, the Institute of Tropical Medicine Antwerp, and many other institutions across south-east Asia, India, and Africa. This ability to forge research partnerships around the world was critical to the success of the clinical development programme.

In 2004, when MMV, Sigma-Tau, the Holley group, and the University of Oxford first signed a contract to cover the clinical development of DHA-PQP to international GMP standards, their partnership was perceived as unlikely to succeed because of the diverse and sometimes conflicting priorities of the individual partners. Nevertheless, a not-for-profit PDP foundation and a mid-sized Italian pharmaceutical company successfully combined their respective strengths to respond to an urgent public health need for new anti-malarial treatments of high quality. Clearly, not all of the objectives of the individual partners were the same, but through this common goal, both partners possessed the resolve to work together to overcome the hurdles encountered along the way to achieving EMA registration for Eurartesim, and they produced a partnership that was greater than the sum of its individual parts.

The development of Eurartesim*,* co-funded by MMV and Sigma-Tau, is a good example of critical research driven by generosity and vision, both in terms of the individual partners involved, and the broader malaria community. It represents an important innovation for patients in resource-poor settings, especially where transmission is high. Poor-quality anti-malarial drugs can lead to drug resistance and inadequate treatment, and they pose an urgent threat in such patient populations [1]. In addition to Eurartesim, several other forms of ACT are available for use by public health systems in malaria-endemic countries. This is important, as by providing a range of therapies to treat malaria, disease-endemic countries can adapt control strategies to their specific needs. The use of multiple first-line therapies against malaria may also delay drug resistance [33,34].

Additionally, studies have demonstrated that DHA-PQP is efficacious against the blood-stage of *P. vivax* malaria [35], however, to date it has only been approved for treatment of *P. falciparum*. Preliminary evidence also indicates that DHA-PQP is one of two currently available formulations of ACT potentially able to improve the anti-relapse effect of primaquine [36,37]. Further studies will be required to confirm these two indications.

Achievement of EMA marketing authorization for Eurartesim does not guarantee its access. In fact, the next objective is to ensure successful uptake of Eurartesim by public health systems in malaria-endemic countries. This poses a number of new challenges, which include ensuring local registration, successful launch, affordable pricing and distribution in these countries, and operational introduction into national malaria control programmes. MMV will clearly need additional new partnerships to address these challenges, the key to which will involve ensuring a win-win solution for partners with differing objectives. MMV has already worked with Sigma-Tau’s expert packaging team to design appropriate packaging for Eurartesim suitable for use by public health systems in countries with low literacy levels, and both partners have decided to continue their partnership to ensure the monitoring of the long-term safety and efficacy of Eurartesim.

Since July 2012, more than 400,000 packages of Eurartesim have been delivered to Cambodia – the first shipments to a malaria-endemic country. This is particularly significant as Cambodia is situated at the epicentre of emerging resistance to many existing anti-malarials. Cambodia had adopted DHA-PQP as first-line treatment for malaria and was awaiting approval by a stringent regulatory agency to allow procurement of Eurartesim using international donor funds. Cambodia has now registered Eurartesim. An agreed price of USD 0.66-1.98 per dose (depending on weight) was accepted by the Global Fund for purchase through The Affordable Medicines Facility–malaria [38]. The first procurement was achieved through a collaboration between the Ministry of Health Cambodia (National Center for Malaria Control, Parasitology and Entomology and the Department of Drugs and Food), Population Services International (PSI; Cambodia and Kenya), WHO (Geneva and Cambodia), Sigma-Tau, and MMV using funds from the Global Fund via the Affordable Medicines Facility–malaria.

Sigma-Tau has recently submitted the Eurartesim dossier for WHO prequalification. Additionally, it has been registered in Ghana Tanzania and Burkina Faso and submitted for registration in Mozambique where a multicentre, multicountry Phase IV study is planned to monitor the effectiveness and safety of new, approved anti-malarial drugs in “real-life” settings. This is part of the risk management plan agreed with the EMA and will allow for the long-term follow-up of the medicine.

In addition, the MMV–Sigma-Tau partnership is currently developing a paediatric dispersible formulation of Eurartesim suitable for use by children between the ages of six months and five years, which will remain stable in hot and humid conditions. This dispersible formulation will improve ease of use by eliminating the need to crush the tablets before administration. A recent meta-analysis has indicated that a higher dose of piperaquine could increase the efficacy of the combination for children aged between one and five years [39], however, the safety of an increased dose in this population has not been evaluated. A complete risk–benefit analysis will need to be made before a change in dosage could be considered. The dosage for the paediatric formulation will be based on EMA recommendations for this age group. Completion of the development programme and preparation of the dossier for submission to the EMA is planned by the end of 2014.

## Conclusion

Together, MMV and Sigma-Tau have been able to develop a safe and efficacious DHA-PQP combination medicine to internationally-recognized quality standards, providing a safety statement to guide its correct use. As a result the first shipment of Eurartesim to a malaria-endemic country was made in July 2012 to Cambodia. The development of Eurartesim exemplifies the strengths of the PDP model in utilising the individual skills and expertise of partners with differing objectives to achieve a common goal. The full partnership extended beyond MMV and Sigma-Tau, and included many collaborators around the world. Ensuring access to Eurartesim in malaria-endemic countries will require the formation of new additional partnerships in order to overcome the challenges involved in successfully launching and distributing the product, as well as its operational introduction into national malaria control programmes. MMV and Sigma-Tau will continue their work together to achieve EMA registration of the paediatric formulation of Eurartesim in the fastest possible time.

## Competing interests

At the time of writing DU and EP were employed by MMV who co-funded the development of Eurartesim^®^.

## Authors’ contributions

DU, as a Director of Clinical Development at MMV, contributed to the strategic development plan, design of the clinical studies and the regulatory submission of Eurartesim. EP, as Editor and Publication’s Officer at MMV, coordinated and contributed to the drafting, editing and review of the article. Both authors read and approved the final manuscript.

## Supplementary Material

Additional file 1 Eurartesim^®^ (dihydroartemisinin-piperaquine) safety statement. Click here for file
