# Supplementary material for: MMV in partnership: the Eurartesim® experience
Source: Malar J. 2013 Jun 19;12:211. doi: 10.1186/1475-2875-12-211 (PMC3691732; doi:10.1186/1475-2875-12-211)
Supplement: Additional file 1 — Eurartesim® (dihydroartemisinin-piperaquine) safety statement. [file 1475-2875-12-211-S1.docx]

**Eurartesim® (dihydroartemisinin-piperaquine) safety statement**

Dihydroartemisinin-piperaquine is generally well tolerated. Its safety has been evaluated in two open-label studies in which it was used to treat 1239 paediatric patients aged up to 18 years and 566 adults aged over 18 years.

Side-effects were found to be mild and generally not serious. In adults, the most common side-effects (seen in between one and 10 patients in 100) are anaemia (low haemoglobin concentration), headache, QTc prolongation (an alteration of the electrical activity of the heart, which can cause a life-threatening abnormality of heart rhythm), tachycardia (rapid heartbeat), asthenia (weakness) and pyrexia (fever). In children, the most common side-effects (seen in more than one patient in 10) were influenza (flu), cough and pyrexia.

Dihydroartemisinin-piperaquine must not be used in patients who are hypersensitive (allergic) to the active substances or any of the other ingredients. It must not be used in patients with severe malaria (which may be life-threatening). It must not be used in patients who have or are at risk of QTc interval prolongation or cardiac arrhythmias (unstable heartbeat) due to heart conditions or taking medicines that can affect heart rhythm. Due to this risk of QTc prolongation it is important that *Eurartesim* is taken without food and at least three hours from any meal. Dihydroartemisinin-piperaquine should also not be used during pregnancy if an effective alternative medicine is available.
